# Supplementary material for: Microbiome and pediatric leukemia, diabetes, and allergies: Systematic review and meta-analysis
Source: PLoS One. 2025 May 20;20(5):e0324167. doi: 10.1371/journal.pone.0324167 (PMC12091780; doi:10.1371/journal.pone.0324167)
Supplement: S1 File — (PDF) [file pone.0324167.s001.pdf]

Supplemental Materials

Table of Contents

S1 Table. Relative abundance of bacterial genera in cases compared to controls by disease type ……………………………………2

S2 Table. Quality assessment for meta-analyses…………………………………………………………………………...12

S1 Figure. Funnel plots assessing publication bias in meta-analyses………………………………………………………13

S2 Figure. Alpha diversity (Shannon Index) in ALL cases compared to controls without antibiotic exposure……………………...…14

S3 Figure. Alpha diversity (Shannon Index) in T1DM cases compared to controls without high-risk haplotype………………………14

S4 Figure. Alpha diversity (Shannon Index) in T1DM cases compared to controls prior to treatment………………………...…….14

S5 Figure. Alpha diversity (Shannon Index) in T1DM cases compared to controls at the time of diagnosis…………………………...15

S6 Figure. Alpha diversity (Shannon Index) in eczema cases compared to controls at or before diagnosis……………………………15

S7 Figure. Alpha diversity (Shannon Index) in eczema cases compared to controls without probiotic exposure………………………16

S8 Figure. Alpha diversity (Shannon Index) in atopy cases compared to controls at or before diagnosis………………………………16

S9 Figure. Alpha diversity (Shannon Index) in atopy cases compared to controls without probiotic exposure……………………..…..16

S10 Figure. Alpha diversity (Shannon Index) in atopy cases compared to controls in studies without family history of allergies as inclusion criterion …………………………………………………………………………………………………......17

S11 Figure. Alpha diversity (Shannon Index) in food allergy cases compared to controls in studies without family history of allergies as inclusion criterion…………………………………………………………………………………………………..17

S12 Figure. Alpha diversity (Shannon Index) in food allergy cases compared to controls at or before diagnosis……………………...17

S1 Appendix. Systematic Review and Meta-analysis Protocol ……………………………………………………..………...……...18

S2 Appendix. Search Terms by Database ………………………………………………...…………………………………...…20

S3 Appendix. List of variables collected in data extraction ………………………………………………………………...…...23

References………..24

S1 Table. Relative abundance of bacterial genera in cases compared to controls by disease type

	Author (Year)	Time	ALL	T1DM	Eczema	Asthma	Atopy	Food allergy
Lactobacillus	Gao (2020)	Diagnosis	↓					
	Biassoni (2020)	Diagnosis		↓				
	Murri (2013)	7 yrs		↓				
	Liwen (2018)					=		
	Zheng (2022)	Enrollment				↑		
	Abdelhamid (2022)	Enrollment				↓		
	Tang (2016)	1 mo			↑			
	West (2015)	1 mo			↑			
	Chan (2020)	4 mo			↓			
	Abrahamsson (2012)	12 mo			=			
	VanNimwegen (2011)	1 mo			↑		↓	↑
	Bjorksten (1999)						↓	
	Suzuki (2008)	6 mo					↑	
Prevotella	Gao (2020)	Diagnosis	↓					
	Chua (2020)	Diagnosis	↓					
	Harbison (2019)	Diagnosis		↓				
	Leiva-Gea (2018)	5 yr from diagnosis		↑				
	Murri (2013)	7 yrs		↓				
	Mejia Leon (2014)	Diagnosis		↓				
	Singh (2021)			↓				
	Abrahamsson (2012)	12 mo			↑			
	Tang (2016)	1 mo			↑			
	Chan (2020)	4 mo			↑			
	Kim (2022)	6 mo – 1 yr			↑			
	Mahdavinia (2017)				↓			
	Zheng (2022)	At enrollment				↓		
	Simonyte Sjodin (2019)	6 mo					↓	
	Drell (2015)	5 yr					↑	
	Chen (2016)	6-24 mo						↓
	Kanchongkittiphon (2024)	At enrollment						↑

Atopobium	Chua (2020)	Diagnosis	↓					
	Chan (2020)	4 mo			↑			
	Holgerson (2023)	3mo, 5yr					↑	
	Joseph (2022)	6 mo						↑
Escherichia-Shigella	Rajagopala (2020)	Diagnosis	↓					
	Yaun (2022)	Diagnosis		↑				
	West (2015)	1 mo			↓			
	Song (2016)	Enrollment			↓			
	Yan (2023)	0-3 yr						↑
Shigella	Tang (2016)	1 mo			↓			
Escherichia	Cinek (2018)	Diagnosis		↑				
	Tang (2016)	1 mo			↓			
	Chan (2020)	4 mo			↓			
	Hoskinson (2023)	1 yr			↑			
	VanNimwegen (2011)	1 mo			↑	↑	↑	↓
	Chiu (2020)	Enrollment				↑		
	Wan (2023)						↑	
	Hara (2024)	17 – 19 mo						↑
	Inoue (2017)	Enrollment						↑
Bifidobacterium	Gao (2020)	Diagnosis	↓					
	Chua (2020)	Diagnosis	↓					
	Leiva-Gea (2018)	5 yr from diagnosis		↓				
	Traversi (2020)	Diagnosis		↓				
	Murri (2013)	7 yrs		↓				
	Cinek (2017)	9, 12, & 15 month (composite)		↓				
	Tang (2016)	1 mo			↓			
	West (2015)	1 mo			↓			
	Abrahamsson (2012)	12 mo			↓			
	Reddel (2019)	Enrollment			↓			
	Chan (2020)	4 mo			↑			
	Mahdavinia (2017)	Enrollment			↑			
	Mah (2006)	3 yr			↓			

Bifidobacterium (Cont.)	Sung (2022)	12 mo			↓			
	Kim (2022)	6 mo – 1 yr			↑			
	VanNimwegen (2011)	1 mo			↑	↓		
	Liwen (2018)	Enrollment				↓		
	Chiu (2023)	Diagnosis				↓		
	Wan (2023)					↑		
	Arrieta (2015)	12 mo					↓	
	Simonyte-Sjodin (2019)	6 mo					↑	
	Arrieta (2018)	3 mo					↑	
	Bjorksten (1999)						↑	
	Kalliomaki (2001)	3 wk					↓	
	Suzuki (2008)	6 mo					↑	
	Drell (2015)	5 yr					↑	
	Holgerson (2023)	3 mo, 5 yr					↑	
	Inoue (2017)	Enrollment						↓
	Yan (2023)	0 – 3 yr						↓
	Castro (2024)	< 1 yr						↓
	De Paepe (2024)							↓
	Joseph (2022)	6 mo						↑
Bacteroides	Rajagopala (2020)	Diagnosis	↑					
	Gao (2020)	Diagnosis	↓					
	Chua (2020)	Diagnosis	↑					
	Leiva-Gea (2018)	5 yr from diagnosis		↑				
	Traversi (2020)	Diagnosis		↓				
	Murri (2013)	7 yrs		↑				
	Mejia Leon (2014)	Diagnosis		↑				
	Singh (2021)	Enrollment		↑				
	Cinek (2017)	9, 12, & 15 month (composite)		↓				
	Belteky (2023)	1 yr		↑				
	Yaun (2022)	Diagnosis		↑				
	Tang (2016)	1 mo			↑			
	West (2015)	1 mo			↑			
	Abrahamsson (2012)	12 mo			↓			
	Reddel (2019)	Enrollment			↑			
	Chan (2020)	4 mo			↓			

Bacteroides (Cont.)	Mahdavinia (2017)	Enrollment			↓			
	Nylund (2013)	6 mo & 18 mo			↓			
	Sung (2022)	12 mo			↑			
	VanNimwegen (2011)	1 mo			↑	↑		
	Wan (2023)					↓		
	Zheng (2022)	Enrollment				↓		
	Chen (2023)	1 yr				↓		
	Holgerson (2023)	3 mo, 5 yr					↓	
	Wan (2023)						↓	
	SimonyteSjodin (2019)	6 mo					↓	
	Bjorksten (1999)						=	
	Kalliomaki (2001)	3 wk					↑	
	Suzuki (2008)	6 mo					↑	
	Drell (2015)	5 yr					=	
	VanNimwegen (2011)	1 mo					↑	↓
	Chen (2016)	6-24 mo						↓
	Hara (2024)	17 – 19 mo						↓
	De Paepe (2024)							↓
	Chen (2024)	Enrollment						↓
	Inoue (2017)	Enrollment						↑
Megamonas	Rajagopala (2020)	Diagnosis	↑					
	Mejia-Leon (2014)	Diagnosis		↓				
	Chan (2020)	4 mo			↓			
	Zheng (2022)	Enrollment				↓		
	Shen (2019)	6 mo					↓	
	Kanchongkittiphon (2024)	Enrollment						↓
Roseburia	Rajagopala (2020)	Diagnosis	↓					
	Gao (2020)	Diagnosis	↓					
	Leiva-Gea (2018)	5 yr from diagnosis		↓				
	Cinek (2018)	Diagnosis		↓				
	Xu (2022)	Diagnosis		↓				
	Belteky (2023)	1 yr		↓				
	Yaun (2022)	Diagnosis		↓				
	Tang (2016)	1 mo			↓			
	Mahdavinia (2017)	Enrollment			↑			
	Patrick (2020)	1 yr				↓		

Roseburia (Cont.)	Petersen (2021)	3 mo					↓	
	Shen (2019)	1 mo					↑	
	Inoue (2017)	Enrollment						↑
	Fazlollahi (2018)	Enrollment						↑
Enterococcus	Gao (2020)	Diagnosis	↑					
	Murri (2013)	7 yrs		↑				
	Endesfelder (2014)	6 mo		↑				
	Belteky (2023)	1 yr		↑				
	Tang (2016)	1 mo			↑			
	West (2015)	1 mo			↓			
	Abrahamsson (2012)	12 mo			↑			
	Reddel (2019)	Enrollment			↓			
	Chan (2020)	4 mo			↓			
	Mah (2006)	3 yr			↑			
	Hoskinson (2023)	1 yr			↑			
	SimonyteSjodin (2019)	6 mo					↑	
	Bjorksten (1999)						↑	
	Joseph (2022)	6 mo						↑
	Chen (2016)	6-24 mo						↑
Staphylococcus	Chua (2020)	Diagnosis	↓					
	Tang (2016)	1 mo			↑			
	West (2015)	1 mo			↓			
	Reddel (2019)	Enrollment			↓			
	Suzuki (2008)	6 mo					↑	
	Holgerson (2023)	3 mo, 5 yr					↑	
Streptococcus	Leiva-Gea (2018)	5 yr from diagnosis		↑				
	Kostic (2015)	Diagnosis		↑				
	Tang (2016)	1 mo		↑				
	Mokhtari (2023)	Enrollment		↑				
	Chierico (2022)	Diagnosis		↓				
	Park (2020)	6 mo			↑			
	West (2015)	1 mo			↓			
	Abrahamsson (2012)	12 mo			↓			
	Chan (2020)	4 mo			↓			
	Wan (2023)					↑	↑	

Streptococcus (Cont.)	Holgerson (2023)	3 mo, 5 yr					↑	
	Vu (2021)	3 mo					↓	
	Arrieta (2018)	3 mo					↓	
	Petersen (2021)	3 mo					↓	
	Bjorksten (1999)						↓	
	Joseph (2022)	6 mo						↑
	Chen (2016)	6-24 mo						↓
Clostridium	Rajagopala (2020)	Diagnosis	↓					
	Murri (2013)	7 yrs		↑				
	Xu (2022)	Diagnosis		↑				
	Chierico (2022)	Diagnosis		↓				
	Singh (2021)	Enrollment		↓				
	Tang (2016)	1 mo			↑			
	Park (2020)	6 mo			↓			
	West (2015)	1 mo			↓			
	Abrahamsson (2012)	12 mo			↓			
	Chan (2020)	4 mo			↓			
	Mahdavinia (2017)	Enrollment			↓			
	Mah (2006)	3 yr			↓			
	Sung (2022)	12 mo			↓			
	Kim (2022)	6 mo – 1 yr			↓			
	Nylund (2013)	6 & 18 mo			↑			
	VanNimwegen (2011)	1 mo			↑	↑	↑	
	SimonyteSjodin (2019)	6 mo					↑	
	Arrieta (2018)	3 mo					↓	
	Bjorksten (1999)						↑	
	Kalliomaki (2001)	3 wk					↑	
	Suzuki (2008)	6 mo					↑	
	Low (2017)	3 mo					↓	
	Holgerson (2023)	3 mo, 5 yr					↓	
	VanNimwegen (2011)	1 mo						↑
	Chen (2024)	Enrollment						↑
	Chen (2016)	6-24 mo						↓
	Savage (2018)	3-6 mo						↓
	Yan (2023)	0 – 3 yr						↓

Klebsiella	Tang (2016)	1 mo			↑			
	Chan (2020)	4 mo			↑			
	Fan (2022)	6 mo			↑			
	Low (2017)	3 mo					↑	
	Wan (2023)						↑	
Haemophilus	Marrs (2021)	3-4 mo			↑			
	Savage (2018)	3-6 mo						↓
Enterobacter	Leiva-Gea (2018)	5 yrs from diagnosis		↑				
	Endesfelder (2014)	2 yr		↑				
	Tang (2016)	1 mo			↑			
	Cheung (2023)	6 mo			↑			
Veillonella	Gao (2020)	Diagnosis	↑					
	Chua (2020)	Diagnosis	↓					
	Leiva-Gea (2018)	5 yrs from diagnosis		↑				
	Murri (20130	7 yrs		↑				
	Tang (2016)	1 mo			↓			
	Abrahamsson (2012)	12 mo			↓			
	Chan (2020)	4 mo			↑			
	Stiemsma (2016)	12 mo				↑		
	Arrieta (2015)	12 mo					↓	
	Arrieta (2018)	3 mo					↓	
	Suzuki (2008)	6 mo					↑	
	Chen (2016)	6-24 mo						↓
	Inoue (2017)	Enrollment						↑
	De Paepe (2024)	Enrollment						↑
Blautia	Rajagopala (2020)	Diagnosis	↑					
	Gao (2020)	Diagnosis	↓					
	Leiva-Gea (2018)	5 yr from diagnosis		↑				
	Kostic (2015)	Diagnosis		↑				
	Belteky (2023)	1 yr		↑				
	Xu (2022)	Diagnosis		↓				
	Yaun (2022)	Diagnosis		↓				

Blautia (Cont.)	Reddel (2019)	Enrollment			↓			
	Hoskinson (2023)	1 yr			↓			
	Mahdavinia (2017)	Enrollment			↑			
	Wan (2023)					↑		
	SimonyteSjodin (2019)	6 mo					↑	
	Arrieta (2018)	3 mo					↓	
	Shen (2019)	1 mo					↑	
	Chen (2016)	6-24 mo						↓
	Hoskinson (2023)	1 yr						↓
	Joseph (2022)	6 mo						↓
	De Paepe (2024)							↓
	Yan (2023)	0 – 3 yr						↑
Eubacterium	Rajagopala (2020)	Diagnosis	↓					
	Cinek (2018)	Diagnosis		↓				
	Xu (2022)	Diagnosis		↑				
	Reddel (2019)	Enrollment			↓			
	Hoskinson (2023)	1 yr			↓			
	Zheng (2022)	Enrollment				↓		
	Petersen (2021)	3 mo					↓	
	Bjorksten (1999)						=	
	Suzuki (2008)	6 mo					↑	
	Hoskinson (2023)	1 yr					↓	↓
	Inoue (2017)	enrollment						↓
Coprococcus	Xu (2022)	Diagnosis		↓				
	Mokhtari (2023)	Enrollment		↑				
	Reddel (2019)	Enrollment			↓			
	SimonyteSjoden (2019)	6 mo					↓	
	Arrieta (2018)	3 mo					↓	
	Wan (2023)						↑	
	Chen (2016)	6-24 mo						↓
Oscillospira	Mahdavinia (2017)	Enrollment			↓			
	Inoue (2017)	Enrollment						↓
	Savage (2018)	3-6 mo						↓

Akkermansia	Rajagopala (2020)	Diagnosis	↑					
	Singh (2021)	Enrollment		↑				
	Chierico (2022)	Diagnosis		↓				
	Park (2020)	6 mo			↓			
	Abrahamsson (2012)	12 mo			=			
	Chan (2020)	4 mo			↓			
	Petersen (2021)	3 mo					↓	
	Drell (2015)	5 yr					↓	
	Inoue (2017)	Enrollment						↓
Dialister	Rajagopala (2020)	Diagnosis	↓					
	Gao (2020)	Diagnosis	↓					
	Chierico (2022)	Diagnosis		↓				
	Xu (2022)	Diagnosis		↓				
	Mahdavinia (2017)	Enrollment			↓			
	Drell (2015)	5 yr					↑	
	Savage (2018)	3 – 6 mo						↓
	Inoue (2017)	Enrollment						↑
Faecalibacterium	Rajagopala (2020)	Diagnosis	↑					
	Gao (2020)	Diagnosis	↓					
	Leiva-Gea (2018)	5yr after diagnosis		↓				
	Xu (2022)	Diagnosis		↓				
	Abrahamsson (2012)	1 yr			↓			
	Song (2016)	Enrollment			↑			
	Reddel (2019)	Enrollment			↑			
	Mahdavinia (2017)	Enrollment			↑			
	Chiu (2020)	Enrollment				↓	↓	
	Drell (2015)	5 yr					=	
	Simonyte Sjodin (2019)	6 mo					↑	
	Arrieta (2015)	12 mo					↑	
	Inoue (2017)	Enrollment						↓
	Fazlollahi (2018)	Enrollment						↑

Lachnospira	Rajagopala (2020)	Diagnosis	↓					
	Leiva-Gea (2018)	5yr after diagnosis		↓				
	Xu (2022)	Diagnosis		↓				
	Stiemsma (2016)	3 mo				↓		
	Arrieta (2015)	12 mo					↓	
	Petersen (2021)	3 mo					↓	
	Chen (2016)	Enrollment						↑
Parabacteroides	Rajagopala (2020)	Diagnosis	↑					
	Tang (2016)	1 mo			↑			
	Song (2016)	Enrollment			↑			
	Abrahamsson (2012)	12 mo			=			
	Reddel (2019)	Enrollment			↑			
	Chan (2020)	4 mo			↓			
	Nylund (2013)	6 mo, 18 mo			↓			
	Drell (2015)	5 yr					=	
	Inoue (2017)	Enrollment						↓
Ruminococcus	Rajagopala (2020)	Diagnosis	↑					
	Gao (2020)	Diagnosis	↓					
	Leiva-Gea (2018)	5 yr after diagnosis		↑				
	Kostic (2015)	Diagnosis		↑				
	Xu (2022)	Diagnosis		↓				
	Song (2016)	Enrollment			↑			
	Abrahamsson (2012)	12 mo			↑			
	Chan (2020)	4 mo			↑			
	Mahdavinia (2017)	Enrollment			↑			
	Nylund (2013)	6 mo, 18 mo			↑			
	SimonyteSjodin (2019)	6 mo					↓	
	Arrieta (2018)	3 mo					↓	
	Chua (2018)						↑	
	Inoue (2017)	Enrollment						↑
	Fazlollahi (2018)	Enrollment						↑
	Azad (2015)	3 mo						=

↓ represents lower relative abundance in cases compared to controls (dark orange = lower relative abundance and p<0.05, light orange = lower relative abundance and p>0.05). ↑ represents higher relative abundance in cases compared to controls (dark green = higher relative abundance and p<0.05, light green = higher relative abundance and p>0.05). = (light blue) represents no difference in relative abundance in cases compared to controls. Abbreviations: ALL = acute lymphoblastic leukemia, T1DM = type 1 diabetes mellitus

S2 Table. Quality assessment for meta-analyses

Outcome by Disease	Study Design	Number of participants (studies)	Risk of Bias	Inconsistency	Indirectness	Imprecision	Other	SMD (95% CI)	Certainty of the evidence (GRADE)
**ALL:** Alpha diversity	Obs	316 (6 studies)	No serious risk of bias	No serious inconsistency	No serious indirectness	No serious imprecision	Large effect size	-0.78 (-1.21, -0.34)	**Moderate** ⊕⊕⊕◯
**Eczema:** Alpha diversity	Obs	2026 (16 studies)	No serious risk of bias	Serious inconsistency	No serious indirectness	No serious imprecision	Large effect size	-0.34 (-0.56, -0.12)	**Very low** ⊕◯◯◯
**Asthma:** Alpha diversity	Obs	937 (7 studies)	No serious risk of bias	Serious inconsistency	No serious indirectness	Serious imprecision	Large effect size	-0.37 (-0.16, 0.42)	**Very low** ⊕◯◯◯
**T1DM:** Alpha diversity	Obs	1112 (11 studies)	No serious risk of bias	Serious inconsistency	Serious indirectness	No serious imprecision	Large effect size	-1.26 (-3.49, 0.96)	**Very low** ⊕◯◯◯
**Atopy:** Alpha diversity	Obs	1163 (9 studies)	No serious risk of bias	No serious inconsistency	No serious indirectness	No serious imprecision		-0.06 (-0.34, 0.22)	**Low** ⊕⊕◯◯
**FA:** Alpha diversity	Obs	2110 (13 studies)	No serious risk of bias	No serious inconsistency	No serious indirectness	Serious imprecision		-0.11 (-0.63, 0.41)	**Very low** ⊕◯◯◯

Abbreviations: Obs: observational study, SMD: standard mean difference, 95% CI: 95% confidence interval, ALL: acute lymphoblastic leukemia, T1DM: type 1 diabetes mellitus, FA: food allergy

S1 Figure. Funnel plots assessing publication bias in meta-analyses

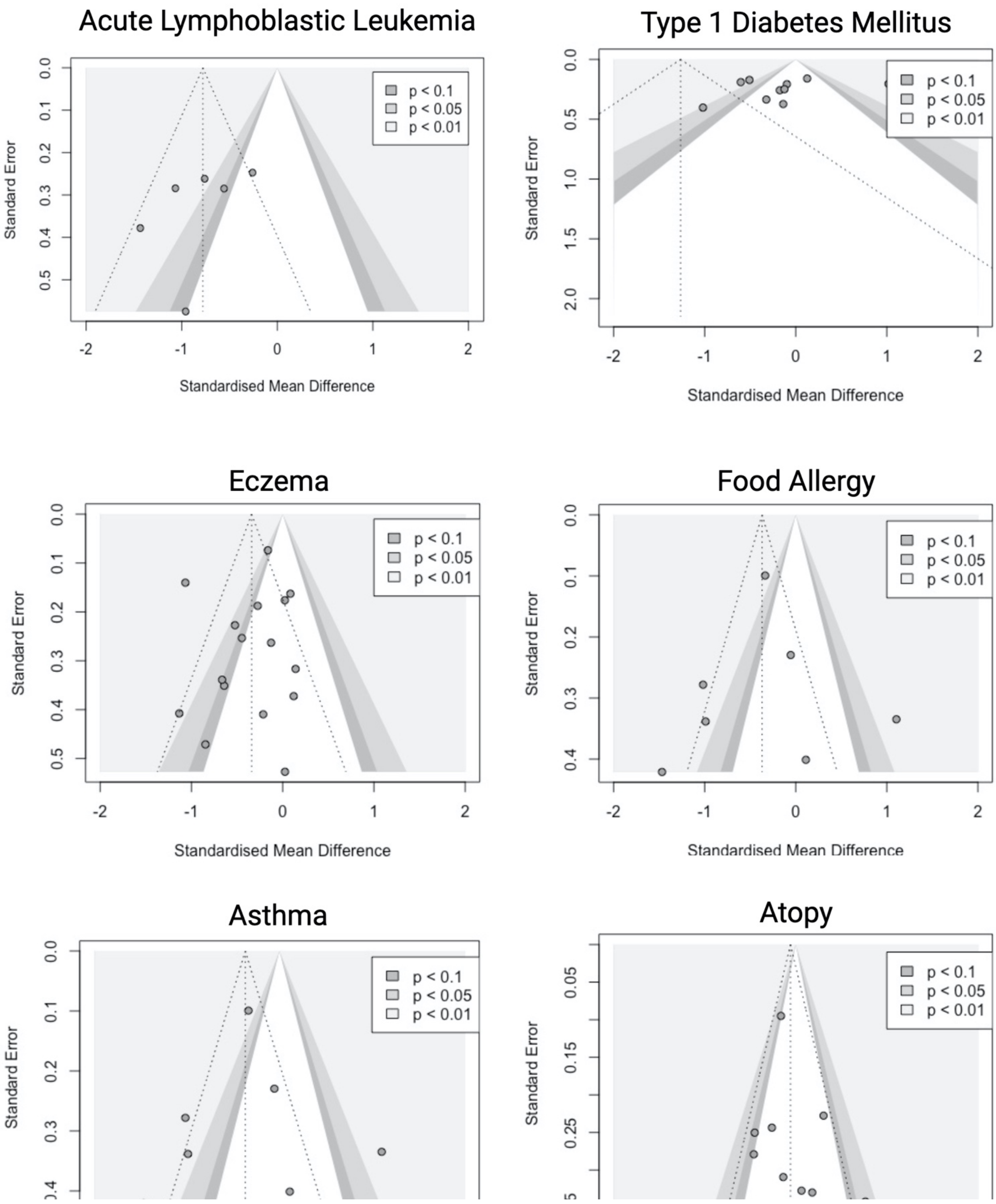

S2 Figure. Alpha diversity (Shannon Index) in ALL cases compared to controls without antibiotic exposure

Study	Experimental Mean	Experimental SD	Experimental Total	Control Mean	Control SD	Control Total	Weight	Std. Mean Difference IV, Random, 95% CI
Gao (2020)	1.42	1.0400	18	2.63	0.5300	18	17.7%	-1.43 [-2.17; -0.69]
Liu (2020)	5.37	0.3800	58	5.47	0.3900	23	25.6%	-0.26 [-0.74; 0.23]
DePietri (2020)	4.57	1.0700	51	5.67	0.8700	19	23.1%	-1.07 [-1.62; -0.51]
Rajagopala (2020)	1.73	0.5500	12	2.23	0.7100	23	18.2%	-0.74 [-1.46; -0.02]
Bai (2017)	2.50	0.7800	10	3.03	0.2300	17	15.5%	-1.02 [-1.86; -0.19]
**Total (95% CI)**			**149**			**100**	**100.0%**	**-0.86 [-1.43; -0.29]**

Heterogeneity: $Tau^2 = 0.1225$; $Chi^2 = 8.77$, df = 4 (P = 0.07); $I^2 = 54\%$

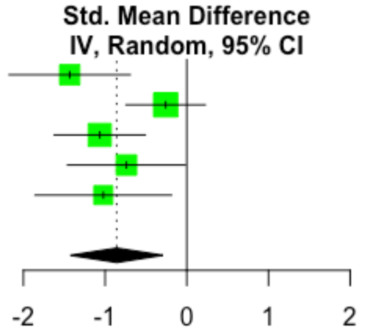

Meta-analysis excluding studies and/or participants with antibiotic exposure.

S3 Figure. Alpha diversity (Shannon Index) in T1DM cases compared to controls without high-risk haplotype

Study	TE	SE	Weight	Std. Mean Difference IV, Random, 95% CI
Chierico (2022)	1.02	0.2038	10.3%	1.02 [0.62; 1.42]
Xu (2022)	-0.60	0.1899	10.3%	-0.60 [-0.98; -0.23]
Belteky (2023)	-0.17	0.2575	10.3%	-0.17 [-0.68; 0.33]
Yuan (2022)	-0.51	0.1718	10.3%	-0.51 [-0.85; -0.17]
Traversi (2020)	-0.10	0.2071	10.3%	-0.10 [-0.50; 0.31]
Leiva-Gea (2018)	-1.02	0.4027	10.2%	-1.02 [-1.81; -0.23]
Singh (2021)	-0.14	0.3731	10.3%	-0.14 [-0.87; 0.59]
Elsherbiny (2022) (Upper Egypt)	-3.64	0.6702	10.0%	-3.64 [-4.96; -2.33]
Elsherbiny (2022) (Lower Egypt)	-14.27	2.1496	7.5%	-14.27 [-18.49; -10.06]
Mokhtari (2023)	-0.12	0.2490	10.3%	-0.12 [-0.61; 0.36]
**Total (95% CI)**			**100.0%**	**-1.61 [-4.47; 1.25]**

Heterogeneity: $Tau^2 = 12.2867$; $Chi^2 = 117.21$, df = 9 (P < 0.01); $I^2 = 92\%$

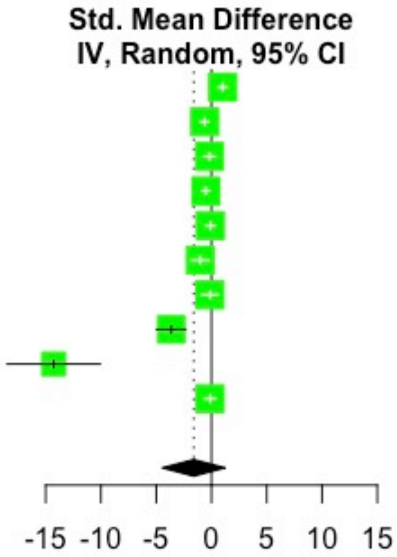

Meta-analysis excluding studies that recruited cases and controls based on high-risk haplotypes for developing type 1 diabetes mellitus.

S4 Figure. Alpha diversity (Shannon Index) in T1DM cases compared to controls prior to treatment

Study	TE	SE	Weight	Std. Mean Difference IV, Random, 95% CI
Chierico (2022)	1.02	0.2038	14.6%	1.02 [0.62; 1.42]
Xu (2022)	-0.60	0.1899	14.9%	-0.60 [-0.98; -0.23]
Belteky (2023)	-0.17	0.2575	13.5%	-0.17 [-0.68; 0.33]
Yuan (2022)	-0.51	0.1718	15.2%	-0.51 [-0.85; -0.17]
Traversi (2020)	-0.10	0.2071	14.5%	-0.10 [-0.50; 0.31]
Vatanen (2018)	0.13	0.1597	15.4%	0.13 [-0.19; 0.44]
Cinek (2017)	-0.32	0.3355	11.8%	-0.32 [-0.98; 0.33]
**Total (95% CI)**			**100.0%**	**-0.07 [-0.58; 0.43]**

Heterogeneity: $Tau^2 = 0.2629$; $Chi^2 = 45.21$, df = 6 (P < 0.01); $I^2 = 87\%$

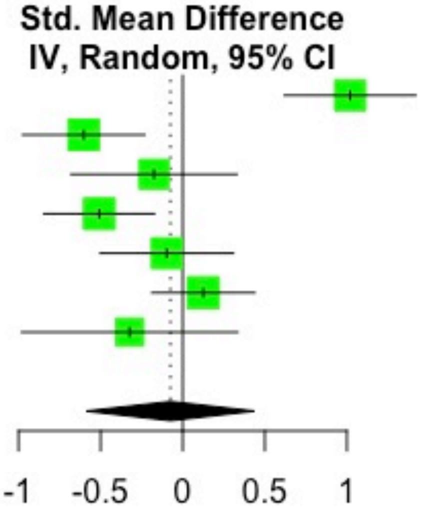

Meta-analysis excluding studies in which microbiome was measured after starting disease directed treatment.

S5 Figure. Alpha diversity (Shannon Index) in T1DM cases compared to controls at the time of diagnosis

Study	TE	SE	Weight	Std. Mean Difference IV, Random, 95% CI
Chierico (2022)	1.02	0.2038	24.8%	1.02 [0.62; 1.42]
Xu (2022)	-0.60	0.1899	25.1%	-0.60 [-0.98; -0.23]
Yuan (2022)	-0.51	0.1718	25.4%	-0.51 [-0.85; -0.17]
Traversi (2020)	-0.10	0.2071	24.8%	-0.10 [-0.50; 0.31]
**Total (95% CI)**			**100.0%**	**-0.05 [-1.23; 1.13]**

Heterogeneity: $Tau^2 = 0.5134$; $Chi^2 = 42.83$, df = 3 ($P < 0.01$); $I^2 = 93\%$

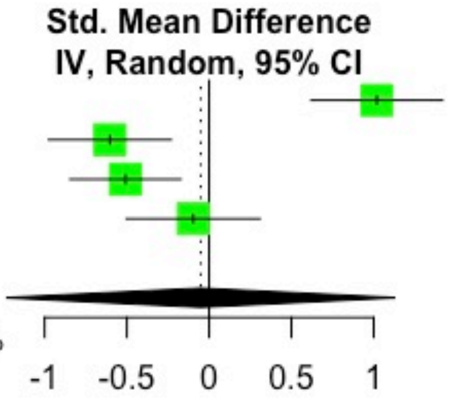

Meta-analysis excluding studies in which microbiome was measured prior to diabetes diagnosis or after starting disease directed treatment.

S6 Figure. Alpha diversity (Shannon Index) in eczema cases compared to controls at or before diagnosis

Study	Experimental Mean	Experimental SD	Experimental Total	Control Mean	Control SD	Control Total	Weight	Std. Mean Difference IV, Random, 95% CI
Lee (2018)	2.80	0.7800	63	2.78	0.8700	66	9.4%	0.02 [-0.32; 0.37]
Tang (2016)	1.38	0.8000	15	1.53	0.4300	10	5.0%	-0.21 [-1.02; 0.59]
Park (2020)	0.77	0.7100	26	1.10	0.6000	84	8.3%	-0.52 [-0.97; -0.08]
Wang (2008)	1.12	0.7300	15	1.48	0.3600	20	5.9%	-0.64 [-1.33; 0.05]
West (2015)	1.66	0.2800	10	1.94	0.3500	10	4.2%	-0.85 [-1.77; 0.08]
Abrahamsson (2012)	2.82	0.8300	20	2.70	0.8300	20	6.5%	0.14 [-0.48; 0.76]
Kang (2021)	2.87	0.4600	61	2.80	0.9800	99	9.7%	0.08 [-0.23; 0.40]
Forno (2008)	0.59	0.3800	9	0.92	0.2500	28	5.0%	-1.13 [-1.93; -0.33]
Patumcharoenpol (2023)	2.75	0.5500	23	2.82	0.5400	39	7.6%	-0.13 [-0.64; 0.39]
Sasaki (2022)	1.57	0.9300	27	1.93	0.6900	39	7.8%	-0.45 [-0.94; 0.05]
Loo (2022)	2.13	0.4500	72	2.67	0.5200	250	10.2%	-1.07 [-1.34; -0.79]
Fan (2022)	2.45	0.6100	10	2.38	0.5500	26	5.6%	0.12 [-0.61; 0.85]
Sung (2023)	2.80	1.2100	6	2.77	1.0500	9	3.6%	0.03 [-1.01; 1.06]
Hoskinson (2023)	2.17	0.3700	282	2.23	0.3700	523	11.3%	-0.16 [-0.31; -0.02]
**Total (95% CI)**			**639**			**1223**	**100.0%**	**-0.33 [-0.58; -0.08]**

Heterogeneity: $Tau^2 = 0.1242$; $Chi^2 = 54.03$, df = 13 ($P < 0.0001$); $I^2 = 75.9\%$

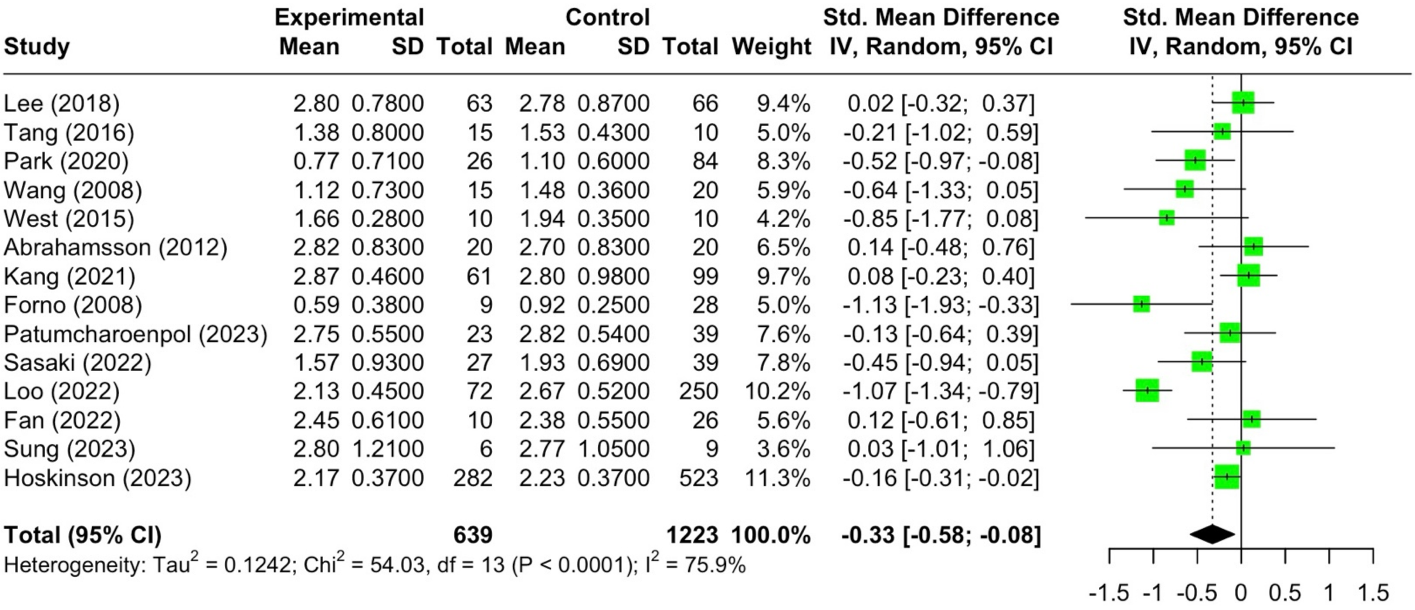

Meta-analysis excluding studies in which microbiome was measured after diagnosis or after initiating treatment of eczema.

S7 Figure. Alpha diversity (Shannon Index) in eczema cases compared to controls without probiotic exposure

	Experimental			Control				Std. Mean Difference
Study	Mean	SD	Total	Mean	SD	Total	Weight	IV, Random, 95% CI
Lee (2018)	2.80	0.7800	63	2.78	0.8700	66	9.3%	0.02 [-0.32; 0.37]
Tang (2016)	1.38	0.8000	15	1.53	0.4300	10	4.7%	-0.21 [-1.02; 0.59]
Park (2020)	0.77	0.7100	26	1.10	0.6000	84	8.1%	-0.52 [-0.97; -0.08]
Wang (2008)	1.12	0.7300	15	1.48	0.3600	20	5.6%	-0.64 [-1.33; 0.05]
West (2015)	1.66	0.2800	10	1.94	0.3500	10	3.9%	-0.85 [-1.77; 0.08]
Song (2016)	0.00	0.3000	90	0.08	0.2700	42	9.0%	-0.27 [-0.64; 0.09]
Kang (2021)	2.87	0.4600	61	2.80	0.9800	99	9.6%	0.08 [-0.23; 0.40]
Forno (2008)	0.59	0.3800	9	0.92	0.2500	28	4.7%	-1.13 [-1.93; -0.33]
Patumcharoenpol (2023)	2.75	0.5500	23	2.82	0.5400	39	7.3%	-0.13 [-0.64; 0.39]
Sasaki (2022)	1.57	0.9300	27	1.93	0.6900	39	7.5%	-0.45 [-0.94; 0.05]
Loo (2022)	2.13	0.4500	72	2.67	0.5200	250	10.1%	-1.07 [-1.34; -0.79]
Fan (2022)	2.45	0.6100	10	2.38	0.5500	26	5.3%	0.12 [-0.61; 0.85]
Sung (2023)	2.80	1.2100	6	2.77	1.0500	9	3.4%	0.03 [-1.01; 1.06]
Hoskinson (2023)	2.17	0.3700	282	2.23	0.3700	523	11.3%	-0.16 [-0.31; -0.02]
**Total (95% CI)**			**709**			**1245**	**100.0%**	**-0.35 [-0.59; -0.12]**

Heterogeneity: $Tau^2 = 0.1100$; $Chi^2 = 52.18$, df = 13 ($P < 0.01$); $I^2 = 75\%$

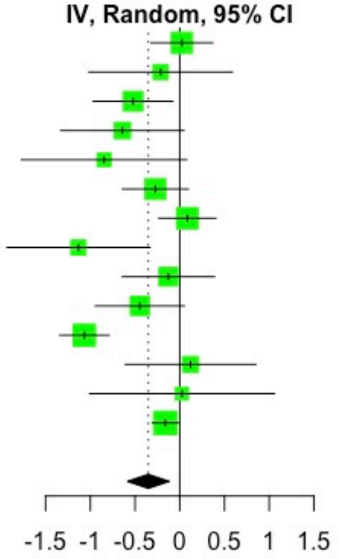

Meta-analysis excluding studies that randomized cases and controls to probiotic treatment versus no probiotic treatment.

S8 Figure. Alpha diversity (Shannon Index) in atopy cases compared to controls at or before diagnosis

	Experimental			Control				Std. Mean Difference
Study	Mean	SD	Total	Mean	SD	Total	Weight	IV, Random, 95% CI
Arrieta (2015)	0.92	0.6700	22	1.13	0.8300	74	10.9%	-0.26 [-0.74; 0.22]
Simonyte-Sjodin (2019)	4.40	0.3000	21	4.48	0.1200	72	10.4%	-0.45 [-0.94; 0.04]
Arrieta (2018)	1.70	0.3000	27	1.57	0.4600	70	12.3%	0.31 [-0.14; 0.75]
Shen (2019)	3.53	0.8100	15	3.40	0.6300	24	6.2%	0.18 [-0.47; 0.83]
Abrahamsson (2014) (SPT)	2.74	0.7400	14	2.69	0.7900	28	6.3%	0.06 [-0.58; 0.71]
Chiu (2020)	5.13	0.7900	20	5.22	0.4800	22	7.0%	-0.14 [-0.74; 0.47]
Hoskinson (2023)	2.17	0.3700	141	2.23	0.3700	523	46.8%	-0.16 [-0.35; 0.02]
**Total (95% CI)**			**260**			**813**	**100.0%**	**-0.11 [-0.32; 0.10]**

Heterogeneity: $Tau^2 = 0.0063$; $Chi^2 = 6.92$, df = 6 ($P = 0.3286$); $I^2 = 13.3\%$

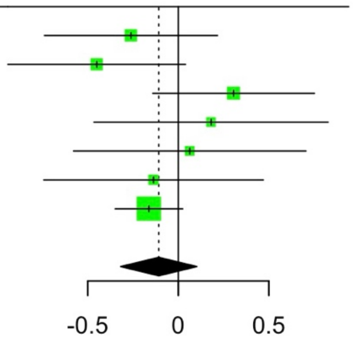

Meta-analysis excluding studies in which microbiome was measured after diagnosis or after initiating treatment of atopy.

S9 Figure. Alpha diversity (Shannon Index) in atopy cases compared to controls without probiotic exposure

	Experimental			Control				Std. Mean Difference
Study	Mean	SD	Total	Mean	SD	Total	Weight	IV, Random, 95% CI
Arrieta (2015)	0.92	0.6700	22	1.13	0.8300	74	14.7%	-0.26 [-0.74; 0.22]
Arrieta (2018)	1.70	0.3000	27	1.57	0.4600	70	15.7%	0.31 [-0.14; 0.75]
Shen (2019)	3.53	0.8100	15	3.40	0.6300	24	10.5%	0.18 [-0.47; 0.83]
Chiu (2020)	5.13	0.7900	20	5.22	0.4800	22	11.4%	-0.14 [-0.74; 0.47]
Hoskinson (2023)	2.17	0.3700	141	2.23	0.3700	523	24.9%	-0.16 [-0.35; 0.02]
Wan (2023)	3.70	0.1600	18	3.48	0.3600	19	10.0%	0.77 [0.09; 1.44]
Chiu (2023)	11.67	3.1400	25	13.33	3.8900	28	12.8%	-0.46 [-1.01; 0.09]
**Total (95% CI)**			**268**			**760**	**100.0%**	**-0.01 [-0.36; 0.34]**

Heterogeneity: $Tau^2 = 0.0635$; $Chi^2 = 12.79$, df = 6 ($P = 0.05$); $I^2 = 53\%$

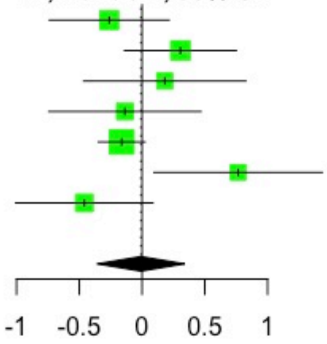

Meta-analysis excluding studies that randomized cases and controls to probiotic treatment versus no probiotic treatment.

S10 Figure. Alpha diversity (Shannon Index) in atopy cases compared to controls in studies without family history of allergies as inclusion criterion

Study	Experimental Mean	Experimental SD	Experimental Total	Control Mean	Control SD	Control Total	Weight	Std. Mean Difference IV, Random, 95% CI
Arrieta (2015)	0.92	0.6700	22	1.13	0.8300	74	12.9%	-0.26 [-0.74; 0.22]
Simonyte-Sjodin (2019)	4.40	0.3000	21	4.48	0.1200	72	12.6%	-0.45 [-0.94; 0.04]
Arrieta (2018)	1.70	0.3000	27	1.57	0.4600	70	13.7%	0.31 [-0.14; 0.75]
Shen (2019)	3.53	0.8100	15	3.40	0.6300	24	9.2%	0.18 [-0.47; 0.83]
Chiu (2020)	5.13	0.7900	20	5.22	0.4800	22	10.0%	-0.14 [-0.74; 0.47]
Hoskinson (2023)	2.17	0.3700	141	2.23	0.3700	523	21.6%	-0.16 [-0.35; 0.02]
Wan (2023)	3.70	0.1600	18	3.48	0.3600	19	8.8%	0.77 [0.09; 1.44]
Chiu (2023)	11.67	3.1400	25	13.33	3.8900	28	11.2%	-0.46 [-1.01; 0.09]
**Total (95% CI)**			**289**			**832**	**100.0%**	**-0.06 [-0.39; 0.26]**

Heterogeneity: $Tau^2 = 0.0656$; $Chi^2 = 14.77$, df = 7 (P = 0.04); $I^2 = 53\%$

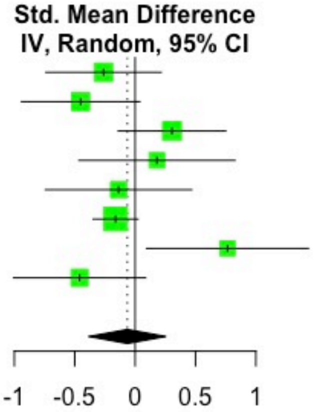

Meta-analysis excluding studies that recruited cases and controls based on family history of allergies.

S11 Figure. Alpha diversity (Shannon Index) in food allergy cases compared to controls in studies without family history of allergies as inclusion criterion

Study	TE	SE	Weight	Std. Mean Difference IV, Random, 95% CI
Inoue (2017)	0.00	0.7071	5.5%	0.00 [-1.39; 1.39]
Kourosh (2018)	0.00	0.3051	8.3%	0.00 [-0.60; 0.60]
Fazlollahi (2018)	0.59	0.1724	9.1%	0.59 [0.25; 0.93]
Azad (2015)	0.24	0.3000	8.4%	0.24 [-0.35; 0.83]
Yan (2023)	-0.47	0.3920	7.8%	-0.47 [-1.24; 0.30]
Hara (2024)	0.61	0.3017	8.4%	0.61 [0.02; 1.21]
Kanchongkittiphon (2024)	-0.36	0.2603	8.6%	-0.36 [-0.87; 0.15]
Yang (2022) (Urban)	-0.18	0.1671	9.1%	-0.18 [-0.51; 0.14]
Yang (2022) (Rural)	0.93	0.2448	8.7%	0.93 [0.45; 1.41]
Hoskinson (2023)	-0.31	0.1095	9.3%	-0.31 [-0.53; -0.10]
Chen (2024)	-2.70	0.3660	7.9%	-2.70 [-3.42; -1.98]
Joseph (2022)	-0.04	0.2092	8.9%	-0.04 [-0.45; 0.37]
**Total (95% CI)**			**100.0%**	**-0.13 [-0.70; 0.45]**

Heterogeneity: $Tau^2 = 0.7032$; $Chi^2 = 96.86$, df = 11 (P < 0.01); $I^2 = 89\%$

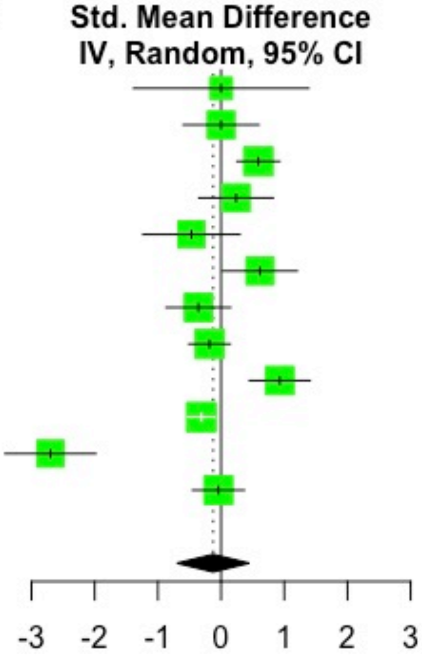

Meta-analysis excluding studies that recruited cases and controls based on family history of allergies.

S12 Figure. Alpha diversity (Shannon Index) in food allergy cases compared to controls in studies at or before diagnosis

Study	SMD	SE	Weight	Std. Mean Difference IV, Random, 95% CI
Azad (2015)	0.2372	0.3000	12.2%	0.24 [-0.35; 0.83]
Savage (2018)	0.0312	0.2812	13.6%	0.03 [-0.52; 0.58]
Yan (2023)	-0.4693	0.3920	7.6%	-0.47 [-1.24; 0.30]
Hoskinson (2023)	-0.3127	0.1095	45.2%	-0.31 [-0.53; -0.10]
Joseph (2022)	-0.0421	0.2092	21.5%	-0.04 [-0.45; 0.37]
**Total (95% CI)**			**100.0%**	**-0.15 [-0.45; 0.14]**

Heterogeneity: $Tau^2 = 0.0168$; $Chi^2 = 4.90$, df = 4 (P = 0.2977); $I^2 = 18.4\%$

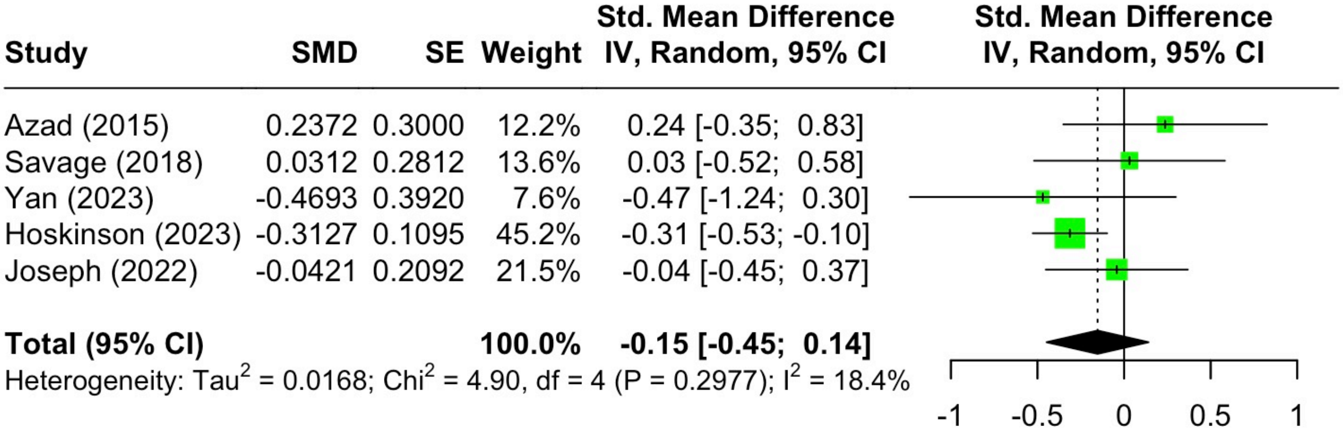

Meta-analysis excluding studies in which microbiome was measured after diagnosis of food allergy.

S1 Appendix. Systematic Review and Meta-analysis Protocol

Characteristics of the microbiome and pediatric acute lymphoblastic leukemia, allergies, and type 1 diabetes: a systematic review and meta-analysis

Rachel Gallant MD MS[1,2,3], Samiha Reza[2], Joseph Wiemels PhD[2], Mel Greaves[3]

[1]Children's Hospital Los Angeles
[2]Center for Genetic Epidemiology, University of Southern California, Keck School of Medicine
3Centre for Evolution and Cancer, Institute of Cancer Research, London, UK

Introduction
Acute lymphoblastic leukemia (ALL) is one of the most common childhood malignancies.[1] With current therapies, most children with ALL achieve a sustained remission, but this does not come without consequences. Treatment for ALL can result in both acute and long-term toxicities affecting survivors into adulthood.[1] Furthermore, diagnosis and treatment of leukemia takes a significant psychological and financial toll on families. For some children, their disease will either relapse or be refractory despite the most sophisticated therapies. For these reasons, the understanding of leukemia etiology is immensely important as it will inform efforts to *prevent* ALL thereby sparing children from the consequences of the disease.

Infection has long been theorized to contribute to ALL development. Specifically, a dysregulated immune response to infection is thought to allow pre-leukemic clones to expand and progress to overt leukemia.[2–4] Children who are exposed to infectious stimuli early in life (such as daycare attendance, vaginal delivery, and breastfeeding) have been shown to have a lower incidence of ALL, suggesting that their immune systems are appropriately primed.[5–7] However, those with delayed infectious exposure may be more likely to have a dysregulated response to infection in childhood and thereby be at higher risk of developing ALL.[2,5] The microbiome is a reflection of these early exposures to infection and has been shown to have a long lasting impact on immune system function.[8,9] Furthermore, there are therapies that can boost the microbiome such as fecal microbiota transplants and administration of probiotics[10–14] making microbiome a modifiable potential risk factor for ALL development.

Childhood allergies and type 1 diabetes have also been linked to a deficiency of gut microbiome.[15–17] These disorders share many of the same risk factors for ALL that reflect gut microbiome. Furthermore, ALL, childhood allergies, and type 1 diabetes have all increased in incidence in recent years, tend to occur in developed countries, and are associated with markers of affluence[18,19] raising the question of whether aberrant microbiome in infancy could be a common predisposing condition for all three of these diseases.

Hypothesis:
Despite the different pathologies and background genetic susceptibilities of these childhood illnesses they all share a common immune priming deficiency contingent upon a lack of a diverse gut microbiome.
If correct, this should be reflected in a consistent sharing of epidemiological risk variables that are proxies for microbiome acquisition and function in early life.

Methods
We will perform a systematic review and meta-analysis on the effect of neonatal microbiome diversity on the development of pediatric ALL, allergies, and type 1 diabetes following PRISMA guidelines. A literature search will be performed using PubMed, Embase, Cochrane, and Web of Science databases. The following search terms will be used in each database: (i) (pediatric AND microbiome AND leukemia) (ii) (pediatric AND microbiome AND allergies) (iii) (pediatric AND microbiome AND type 1 diabetes). A complete list of search terms is included in the Appendix. References will be uploaded into EndNote software and deduplication will be performed systematically.[20,21] References will then be uploaded in Covidence software for screening, review, and data extraction.[22]

Inclusion and exclusion criteria
Case-control studies, meta-analyses, and cohort studies will be considered for inclusion. Studies will be included if the following criteria are met: (i) subjects age 1-18 years, (ii) reports the effect of microbiome in the first year of life (iii) includes outcome of ALL, allergies, asthma, or type 1 diabetes, (iv) published in English. Studies will be excluded based on the following criteria: (i) age < 1 or > 18 years, (ii) outcome of Down Syndrome associated ALL (iii) non-English text, (iv) reviews, pre-print, or abstracts (v) studies with number of cases < 100, (vi) heavily biased studies or those with obvious confounders.

Selection Process
After consultation with a librarian at the University of Southern California Norris Medical Library, two independent reviewers (RG and SR) will perform title and abstract screening of all titles returned using the search strategy outlined above. Full text screening will be performed by independent reviewers. Disagreements will be discussed until a consensus is reached. If needed, a third reviewer will serve as a tie-breaker. Studies not meeting inclusion criteria will be excluded at this point.

Data Extraction Process
Data collection forms will be created in Covidence software.[22] Each study will have data collected by one reviewer. Basic information from each study will be collected including study title, year of publication, study country, study design, study period dates, funding sources, and number of participants. Measures of the microbiome will be collected as either diversity or deficiency of a specific bacterial species. For the former, the metric used will be recorded (e.g. Shannon, Chao1, Simpson, or other measure of diversity), and for the latter the bacterial species of interest will be recorded. We will also collect variables demographic variables including a description of the population, race, and ethnicity. Additional variables to be collected include mode of delivery (vaginal vs c-section), breastfeeding (duration of breastfeeding), antibiotic exposure in infancy (duration or number of antibiotic courses), maternal antibiotic exposure during pregnancy (duration or number of antibiotic courses), exposure to farm animals or pets in infancy, birth order, and daycare attendance (number of hours in daycare per week and number of child contacts per week).

For articles that do not explicitly state the necessary summary statistics for inclusion in meta-analysis, an email will be sent to the corresponding author(s) requesting this information. Authors will respond either by providing the summary statistics or appropriate data to calculate summary statistics or stating that summary statistics for the analysis were no longer in their possession/available. For those authors who do not respond, a second email will be sent requesting summary statistics. If summary statistics are not available in the manuscripts or directly from authors, they will be estimated from graphical depictions of the results as available.

Outcomes
The primary outcome is to identify the effect of microbiome diversity on the risk of pediatric ALL, allergies, and type 1 diabetes; specifically, whether lower microbiome diversity is associated with higher risk of ALL, allergies, and type 1 diabetes. If there are multiple studies that describe a significant association between the deficiency of a specific bacterial species and the diseases of interest, then these studies will undergo a separate analysis to identify the effect of abundance of the bacterial species on risk of pediatric ALL, allergies, and type 1 diabetes. Additionally, we will examine how ethnicity/country of origin, breastfeeding exposure, delivery mode, antibiotic exposure, birth order, and farm animal or pet exposure affect the relationship of microbiome and the diseases of interest by considering these as potential confounders.

Statistical Analysis
The most appropriate summary statistic will be an odds ratio calculated for each of the studies. Weighted estimates will be calculated based on the size of the study. Microbiome diversity will be the predictor variable (using Shannon, Chao1, or Simpson) with disease (ALL, allergies, or type 1 diabetes) as the outcome variable. If there are multiple studies that describe a significant association between the deficiency of a specific bacterial species and the diseases of interest, then these studies will undergo a separate analysis with abundance of the bacterial species as the predictor variable and disease (ALL, allergies, or type 1 diabetes) as the outcome variable. Fixed effect model will be used if the each of the studies are estimating the same quantity. If the estimates of the effect varies across studies, then a random effects model will be used. This analysis will be performed for each disease outcome. Other exposure variables of interest will be considered as confounders such as birth order, breast feeding, delivery mode, antibiotic exposure, daycare exposure, ethnicity, and country of origin. Results will be displayed in forest plots.

S2 Appendix. Search terms by database

PubMed Search

(Pediatrics[MeSH] OR paediatrics OR pediatric OR paediatric OR "paediatric care" OR "pediatric care" OR "paediatric institute" OR "pediatric institute" OR pediatry OR "pediatrics department" OR "paediatrics department")
AND
(Microbiota[MeSH] OR Microbiotas OR "Microbial Community" OR "Microbial Communities" OR "Microbial Community Composition" OR "Microbial Community Compositions" OR Microbiome OR Microbiomes OR "Human Microbiome" OR "Human Microbiomes" OR "Microbial Community Structure" OR "Microbial Community Structures" OR Micro-biome OR "Microbial biome" OR "microbial flora" OR microflora)
AND
("Leukemia"[MeSH] OR leukemias OR leucocythemia OR leucocythemias OR leucocythaemia OR leucocythaemias OR "Leukemia, Lymphoid"[MeSH] OR "precursor cell lymphoblastic leukemia-lymphoma"[MeSH] OR "Leukemias, Lymphoid" OR "Lymphocytic leukemia" OR "lymphocytic leukemias" OR "acute lymphoblastic leukemia" OR "acute lymphoblastic leukaemia" OR "acute lymphatic leukemia" OR "acute lymphatic leukaemia" OR "acute lymphocyte leukaemia" OR "acute lymphocyte leukemia" OR "acute lymphocytic leukaemia" OR "acute lymphocytic leukemia" OR "acute lymphoid leukemia" OR "acute lymphoid leukaemia" OR "B-cell acute lymphoblastic leukemia" OR "B-cell acute lymphoblastic leukaemia" OR "CALLA-positive leukemia" OR "CALLA-positive leukaemia" OR "L1 acute lymphocytic leukaemia" OR "L1 acute lymphocytic leukemia" OR "L2 acute lymphocytic leukaemia" OR "L2 acute lymphocytic leukemia" OR "precursor B-cell lymphoblastic leukaemia-lymphoma" OR "precursor B-cell lymphoblastic leukemia-lymphoma" OR "precursor cell lymphoblastic leukaemia-lymphoma" OR "Leukaemia, Lymphoid" OR "Leukaemias, Lymphoid" OR "Lymphocytic leukaemia" OR "lymphocytic leukemias" OR "lymphoblastic leukemia" OR "lymphoblastic leukaemia" OR "L1 lymphocytic leukemia" OR "L1 lymphocytic leukaemia" OR "acute lymphoblastic leukemia, childhood" OR "L1 acute lymphoblastic leukemia" OR "L1 acute lymphoblastic leukaemia" OR "Childhood ALL" OR "L2 lymphocytic leukemia" OR "L2 lymphocytic leukaemia")
OR
(Hypersensitivity[MeSH] OR "Hypersensitivity, Immediate"[MeSH] OR "Hypersensitivity, Delayed"[MeSH] OR Hypersensitivities OR Allergy OR Allergies OR "Allergic Reaction" OR "Allergic Reactions" OR "Hypersensitivities, Immediate" OR "IgE-Mediated Hypersensitivity" OR "IgE-Mediated Hypersensitivities" OR "Type I Hypersensitivity" OR "Type I Hypersensitivities" OR "Atopic Hypersensitivity" OR "Atopic Hypersensitivities" OR "Hypersensitivity, Type IV" OR "Hypersensitivities, Type IV" OR "Hypersensitivities, Delayed" OR atopy OR atopia OR "atopic allergy" OR "atopic diathesis" OR "atopic disease" OR "atopic skin reaction" OR "atopic syndrome" OR "allergic disease" OR "allergic syndrome" OR allergosis OR "allergic process" OR "allergy type 1" OR "immediate type hypersensitivity" OR "allergy, immediate" OR "immediate type allergy" OR "allergic disease" OR "allergic process" OR "allergic syndrome" OR allergosis OR erethism OR "hyperergic reaction" OR hyperergy OR hypersensibility OR hypersensitiveness OR "immediate type hypersensitivities" OR "immediate hypersensitivity reaction")
OR
("Diabetes Mellitus, Type 1"[MeSH] OR "Diabetes Mellitus, Insulin-Dependent" OR "Diabetes Mellitus, Juvenile-Onset" OR "Diabetes Mellitus, Juvenile Onset" OR IDDM OR "Juvenile-Onset Diabetes" OR "Diabetes Mellitus, Insulin-Dependent,1" OR "Type 1 Diabetes" OR "Diabetes Mellitus, Type I" OR "Diabetes, Autoimmune" OR "Ketosis-Prone Diabetes Mellitus" OR "Brittle Diabetes Mellitus" OR "Ketosis Prone Diabetes Mellitus" OR "Diabetes Mellitus, Insulin Dependent" OR "Diabetes Mellitus, Insulin Dependent, 1" OR "Juvenile Onset Diabetes" OR "brittle diabetes" OR "ketoacidotic diabetes" OR "insulin dependent diabetes" OR "juvenile diabetes" OR "diabetes type 1" OR "juvenile diabetes mellitus" OR "dm 1" OR "early onset diabetes mellitus" OR "diabetes type I" OR T1DM OR "early onset diabetes")

Embase Search

('pediatrics'/exp OR paediatrics OR pediatric OR paediatric OR 'paediatric care' OR 'pediatric care' OR 'paediatric insititute' OR 'pediatric institute' OR 'paediatric practice' OR 'pediatric practice' OR pediatry OR 'pediatrics department' OR 'paediatrics department')
AND
('microflora'/exp OR Microbiotas OR Micro-biome OR 'Microbial biome' OR Microbiota OR 'microbial flora' OR 'microbiome'/exp OR Microbiomes OR 'Microbial Community' OR 'Microbial Communities' OR 'Microbial Community Composition' OR 'Microbial Community Compositions' OR Microbiome OR 'Human Microbiome' OR 'Human Microbiomes' OR 'Microbial Community Structure' OR 'Microbial Community Structures')
AND
('acute lymphoblastic leukemia'/exp OR 'acute lymphoblastic leukaemia' OR 'acute lymphatic leukemia' OR 'acute lymphatic leukaemia' OR 'acute lymphocyte leukaemia' OR 'acute lymphocyte leukemia' OR 'acute lymphocytic leukaemia' OR 'acute lymphocytic leukemia' OR 'acute lymphoid leukemia' OR 'acute lymphoid leukaemia' OR 'B-cell acute lymphoblastic leukemia' OR 'B-cell acute lymphoblastic leukaemia' OR 'CALLA-positive leukemia' OR 'CALLA-positive leukaemia' OR 'L1 acute lymphocytic leukaemia' OR 'L1 acute lymphocytic leukemia' OR 'L2 acute lymphocytic leukaemia' OR 'L2 acute lymphocytic leukemia OR precursor B-cell lymphoblastic leukaemia-lymphoma OR precursor B-cell lymphoblastic leukemia-lymphoma' OR 'precursor cell

lymphoblastic leukemia-lymmphoma' OR 'precursor cell lymphoblastic leukaemia-lymphoma' OR 'Leukemia, Lymphoid' OR 'Leukemias, Lymphoid' OR 'Lymphocytic leukemia' OR 'lymphocytic leukemias' OR 'lymphoblastic leukemia' OR 'lymphoblastic leukaemia' OR 'L1 lymphocytic leukemia' OR 'L1 lymphocytic leukemia' OR 'acute lymphoblastic leukemia, childhood' OR 'L1 acute lymphoblastic leukemia' OR 'L1 acute lymphoblastic leukaemia' OR 'Childhood ALL' OR 'L2 lymphocytic leukemia' OR 'L2 lymphocytic leukaemia')
OR
('hypersensitivity'/exp OR 'atopy'/exp OR 'immediate type hypersensitivity'/exp OR Hypersensitivities OR Allergy OR Allergies OR 'Allergic Reaction' OR 'Allergic Reactions' OR atopia OR 'atopic allergy' OR 'atopic diathesis' OR 'atopic disease' OR 'atopic skin reaction' OR 'Atopic Hypersensitivity' OR 'Atopic Hypersensitivities' OR 'atopic syndrome' OR 'allergic disease' OR 'allergic syndrome' OR allergosis OR 'allergic process' OR 'allergy type 1' OR 'allergy, immediate' OR 'immediate type allergy' OR 'allergic disease' OR 'allergic syndrome' OR allergosis OR erethism OR 'hyperergic reaction' OR 'hyperergy' OR hypersensibility OR hypersensitiveness OR 'immediate type hypersensitivities' OR 'IgE-Mediated Hypersensitivity' OR 'IgE-Mediated Hypersensitivities' OR 'Type I Hypersensitivity' OR 'Type I Hypersensitivities' OR 'Hypersensitivity, Delayed' OR 'Hypersensitivity, Type IV' OR 'Hypersensitivities, Type IV' OR 'Hypersensitivities, Delayed' OR 'immediate hypersensitivity reaction')
OR
('insulin dependent diabetes mellitus'/exp OR 'Diabetes Mellitus, Insulin Dependent' OR 'Diabetes Mellitus, Insulin-Dependent' OR 'Diabetes Mellitus, Juvenile-Onset' OR 'Diabetes Mellitus, Juvenile Onset' OR IDDM OR 'brittle diabetes' OR 'Brittle Diabetes Mellitus' OR 'Type 1 Diabetes' OR 'Diabetes Mellitus Type I' OR 'Diabetes Mellitus Type 1' OR 'Diabetes, Autoimmune' OR 'ketoacidotic diabetes' OR 'insulin dependent diabetes' OR 'juvenile diabetes' OR 'Diabetes Mellitus, Insulin Dependent, 1' OR 'Juvenile Onset Diabetes' OR 'Juvenile-Onset Diabetes' OR 'diabetes type 1' OR 'juvenile diabetes mellitus' OR 'dm 1' OR 'early onset diabetes mellitus' OR 'diabetes type I' OR T1DM OR 'Diabetes Mellitus, Insulin-Dependent,1' OR 'Diabetes, Autoimmune' OR 'Ketosis-Prone Diabetes Mellitus' OR 'Ketosis Prone Diabetes Mellitus' OR 'early onset diabetes')

Cochrane Search

([mh Pediatrics] OR paediatrics OR pediatric OR paediatric OR "paediatric care" OR "pediatric care" OR "paediatric institute" OR "pediatric institute" OR pediatry OR "pediatrics department" OR "paediatrics department")
AND
([mh Microbiota] OR Microbiotas OR "Microbial Community" OR "Microbial Communities" OR "Microbial Community Composition" OR "Microbial Community Compositions" OR Microbiome OR Microbiomes OR "Human Microbiome" OR "Human Microbiomes" OR "Microbial Community Structure" OR "Microbial Community Structures" OR Micro-biome OR "Microbial biome" OR "microbial flora" OR microflora)
AND
([mh Leukemia] OR leukemias OR leucocythemia OR leucocythemias OR leucocythaemia OR leucocythaemias OR [mh "Leukemia, Lymphoid"] OR [mh "precursor cell lymphoblastic leukemia-lymphoma"] OR "Leukemias, Lymphoid" OR "Lymphocytic leukemia" OR "lymphocytic leukemias" OR "acute lymphoblastic leukemia" OR "acute lymphoblastic leukaemia" OR "acute lymphatic leukemia" OR "acute lymphatic leukaemia" OR "acute lymphocyte leukaemia" OR "acute lymphocyte leukemia" OR "acute lymphocytic leukaemia" OR "acute lymphocytic leukemia" OR "acute lymphoid leukemia" OR "acute lymphoid leukaemia" OR "B-cell acute lymphoblastic leukemia" OR "B-cell acute lymphoblastic leukaemia" OR "CALLA-positive leukemia" OR "CALLA-positive leukaemia" OR "L1 acute lymphocytic leukaemia" OR "L1 acute lymphocytic leukemia" OR "L2 acute lymphocytic leukaemia" OR "L2 acute lymphocytic leukemia" OR "precursor B-cell lymphoblastic leukaemia-lymphoma" OR "precursor B-cell lymphoblastic leukemia-lymphoma" OR "precursor cell lymphoblastic leukaemia-lymphoma" OR "Leukaemia, Lymphoid" OR "Leukaemias, Lymphoid" OR "Lymphocytic leukaemia" OR "lymphocytic leukemias" OR "lymphoblastic leukemia" OR "lymphoblastic leukaemia" OR "L1 lymphocytic leukemia" OR "L1 lymphocytic leukaemia" OR "acute lymphoblastic leukemia, childhood" OR "L1 acute lymphoblastic leukemia" OR "L1 acute lymphoblastic leukaemia" OR "Childhood ALL" OR "L2 lymphocytic leukemia" OR "L2 lymphocytic leukaemia")
OR
([mh Hypersensitivity] OR [mh "Hypersensitivity, Immediate"] OR [mh "Hypersensitivity, Delayed"] OR Hypersensitivities OR Allergy OR Allergies OR "Allergic Reaction" OR "Allergic Reactions" OR "Hypersensitivities, Immediate" OR "IgE-Mediated Hypersensitivity" OR "IgE-Mediated Hypersensitivities" OR "Type I Hypersensitivity" OR "Type I Hypersensitivities" OR "Atopic Hypersensitivity" OR "Atopic Hypersensitivities" OR "Hypersensitivity, Type IV" OR "Hypersensitivities, Type IV" OR "Hypersensitivities, Delayed" OR atopy OR atopia OR "atopic allergy" OR "atopic diathesis" OR "atopic disease" OR "atopic skin reaction" OR "atopic syndrome" OR "allergic disease" OR "allergic syndrome" OR allergosis OR "allergic process" OR "allergy type 1" OR "immediate type hypersensitivity" OR "allergy, immediate" OR "immediate type allergy" OR "allergic disease" OR "allergic process" OR "allergic syndrome" OR allergosis OR erethism OR "hyperergic reaction" OR hyperergy OR hypersensibility OR hypersensitiveness OR "immediate type hypersensitivities" OR "immediate hypersensitivity reaction")
OR
([mh "Diabetes Mellitus, Type 1"] OR "Diabetes Mellitus, Insulin-Dependent" OR "Diabetes Mellitus, Juvenile-Onset" OR "Diabetes Mellitus, Juvenile Onset" OR IDDM OR "Juvenile-Onset Diabetes" OR "Diabetes Mellitus, Insulin-Dependent,1" OR "Type 1 Diabetes" OR "Diabetes Mellitus, Type I" OR "Diabetes, Autoimmune" OR "Ketosis-Prone Diabetes Mellitus" OR "Brittle Diabetes

Mellitus” OR “Ketosis Prone Diabetes Mellitus” OR “Diabetes Mellitus, Insulin Dependent” OR “Diabetes Mellitus, Insulin Dependent, 1” OR “Juvenile Onset Diabetes” OR “brittle diabetes” OR “ketoacidotic diabetes” OR “insulin dependent diabetes” OR “juvenile diabetes” OR “diabetes type 1” OR “juvenile diabetes mellitus” OR “dm 1” OR “early onset diabetes mellitus” OR “diabetes type I” OR T1DM OR “early onset diabetes”)

Web of Science Search

(Pediatrics OR paediatrics OR pediatric OR paediatric OR “paediatric care” OR “pediatric care” OR “paediatric institute” OR “pediatric institute” OR pediatry OR “pediatrics department” OR “paediatrics department”)
AND
(Microbiota OR Microbiotas OR “Microbial Community” OR “Microbial Communities” OR “Microbial Community Composition” OR “Microbial Community Compositions” OR Microbiome OR Microbiomes OR “Human Microbiome” OR “Human Microbiomes” OR “Microbial Community Structure” OR “Microbial Community Structures” OR Micro-biome OR “Microbial biome” OR “microbial flora” OR microflora)
AND
(Leukemia OR leukemias OR leucocythemia OR leucocythemias OR leucocythaemia OR leucocythaemias OR “Leukemia, Lymphoid”[MeSH] OR “precursor cell lymphoblastic leukemia-lymphoma”[MeSH] OR “Leukemias, Lymphoid” OR “Lymphocytic leukemia” OR “lymphocytic leukemias” OR “acute lymphoblastic leukemia” OR “acute lymphoblastic leukaemia” OR “acute lymphatic leukemia” OR “acute lymphatic leukaemia” OR “acute lymphocyte leukaemia” OR “acute lymphocyte leukemia” OR “acute lymphocytic leukaemia” OR “acute lymphocytic leukemia” OR “acute lymphoid leukemia” OR “acute lymphoid leukaemia” OR “B-cell acute lymphoblastic leukemia” OR “B-cell acute lymphoblastic leukaemia” OR “CALLA-positive leukemia” OR “CALLA-positive leukaemia” OR “L1 acute lymphocytic leukaemia” OR “L1 acute lymphocytic leukemia” OR “L2 acute lymphocytic leukaemia” OR “L2 acute lymphocytic leukemia” OR “precursor B-cell lymphoblastic leukaemia-lymphoma” OR “precursor B-cell lymphoblastic leukemia-lymphoma” OR “precursor cell lymphoblastic leukaemia-lymphoma” OR “Leukaemia, Lymphoid” OR “Leukaemias, Lymphoid” OR “Lymphocytic leukaemia” OR “lymphocytic leukemias” OR “lymphoblastic leukemia” OR “lymphoblastic leukaemia” OR “L1 lymphocytic leukemia” OR “L1 lymphocytic leukaemia” OR “acute lymphoblastic leukemia, childhood” OR “L1 acute lymphoblastic leukemia” OR “L1 acute lymphoblastic leukaemia” OR “Childhood ALL” OR “L2 lymphocytic leukemia” OR “L2 lymphocytic leukaemia”)
OR
(Hypersensitivity OR “Hypersensitivity, Immediate”[MeSH] OR “Hypersensitivity, Delayed”[MeSH] OR Hypersensitivities OR Allergy OR Allergies OR “Allergic Reaction” OR “Allergic Reactions” OR “Hypersensitivities, Immediate” OR “IgE-Mediated Hypersensitivity” OR “IgE-Mediated Hypersensitivities” OR “Type I Hypersensitivity” OR “Type I Hypersensitivities” OR “Atopic Hypersensitivity” OR “Atopic Hypersensitivities” OR “Hypersensitivity, Type IV” OR “Hypersensitivities, Type IV” OR “Hypersensitivities, Delayed” OR atopy OR atopia OR “atopic allergy” OR “atopic diathesis” OR “atopic disease” OR “atopic skin reaction” OR “atopic syndrome” OR “allergic disease” OR “allergic syndrome” OR allergosis OR “allergic process” OR “allergy type 1” OR “immediate type hypersensitivity” OR “allergy, immediate” OR “immediate type allergy” OR “allergic disease” OR “allergic process” OR “allergic syndrome” OR allergosis OR erethism OR “hyperergic reaction” OR hyperergy OR hypersensibility OR hypersensitiveness OR “immediate type hypersensitivities” OR “immediate hypersensitivity reaction”)
OR
(“Diabetes Mellitus, Type 1” OR “Diabetes Mellitus, Insulin-Dependent” OR “Diabetes Mellitus, Juvenile-Onset” OR “Diabetes Mellitus, Juvenile Onset” OR IDDM OR “Juvenile-Onset Diabetes” OR “Diabetes Mellitus, Insulin-Dependent,1” OR “Type 1 Diabetes” OR “Diabetes Mellitus, Type I” OR “Diabetes, Autoimmune” OR “Ketosis-Prone Diabetes Mellitus” OR “Brittle Diabetes Mellitus” OR “Ketosis Prone Diabetes Mellitus” OR “Diabetes Mellitus, Insulin Dependent” OR “Diabetes Mellitus, Insulin Dependent, 1” OR “Juvenile Onset Diabetes” OR “brittle diabetes” OR “ketoacidotic diabetes” OR “insulin dependent diabetes” OR “juvenile diabetes” OR “diabetes type 1” OR “juvenile diabetes mellitus” OR “dm 1” OR “early onset diabetes mellitus” OR “diabetes type I” OR T1DM OR “early onset diabetes”)

S3 Appendix. List of variables collected in data extraction

- Title of study
- Year of publication
- Lead author contact information
- Country in which study was conducted
- Aim of study
- Study design (cohort, case-control, meta-analysis)
- Start and end date of the study
- Study funding resources
- Possible conflicts of interest for study authors
- Description of the population included in the study
- Inclusion criteria that are met
- Exclusion Criteria that are not met
- Method of recruitment of subjects
- Total number of participants
- Number of cases
- Number of controls
- Microbiome diversity measurement used (Shannon, Chao1, Simpson, etc)
- If microbiome diversity is not measured, then record the bacterial species of significance
- Disease studied: allergies (specify food allergy, seasonal allergy, eczema, etc), asthma, ALL, type 1 diabetes
- Ethnicity if applicable (Latino vs Non-Latino): include proportion/percentages of subjects of each ethnicity
- Race if applicable: include proportion/percentages of subjects of each race
- Delivery mode if applicable (vaginal vs c-section): include proportion/percentages of subjects in each group
- Breastfeeding if applicable: proportion/percentage of cases/controls with breastfeeding exposure AND duration of breastfeeding
- Antibiotic exposure during infancy if applicable: proportion/percentage of cases AND duration
- Maternal antibiotic exposure during pregnancy is applicable: proportion/percentage of cases with antibiotic exposure AND duration
- Exposure to farm animals or pets in infancy if applicable: proportion/percentage of cases/controls exposed AND duration
- Birth order
- Daycare attendance if applicable: proportion/percentage of cases/controls exposed AND numbers of hours per week and/or number of children contacts per week
- Age at diagnosis of disease studied if applicable (median, mean, or range)
- Effect measure reported (OR, HR, etc)
- Effect size
- Lower confidence limit
- Upper confidence limit
- Adjusted variables
- Variables identified as confounders

References

1. Orkin S, Fisher D, Ginsburg D, Look A, Lux S, Nathan D. *Nathan and Oski's Hematology and Oncology of Infancy and Childhood*. Vol 2. 8th ed. Elsevier; 2015.
2. Greaves M. Infection, immune responses and the aetiology of childhood leukaemia. *Nat Rev Cancer*. 2006;6(3):193-203. doi:10.1038/nrc1816
3. Wiemels JL, Cazzaniga G, Daniotti M, et al. Prenatal origin of acute lymphoblastic leukaemia in children. *The Lancet*. 1999;354(9189):1499-1503. doi:10.1016/S0140-6736(99)09403-9
4. Chang JS, Tsai C-R, Tsai Y-W, Wiemels JL. Medically diagnosed infections and risk of childhood leukaemia: a population-based case–control study. *Int J Epidemiol*. 2012;41(4):1050-1059. doi:10.1093/ije/dys113
5. Rudant J, Lightfoot T, Urayama KY, et al. Childhood Acute Lymphoblastic Leukemia and Indicators of Early Immune Stimulation: A Childhood Leukemia International Consortium Study. *Am J Epidemiol*. 2015;181(8):549-562. doi:10.1093/aje/kwu298
6. Gilham C, Peto J, Simpson J, et al. Day care in infancy and risk of childhood acute lymphoblastic leukaemia: findings from UK case-control study. *BMJ*. 2005;330(7503):1294. doi:10.1136/bmj.38428.521042.8F
7. Ma X, Buffler PA, Selvin S, et al. Daycare attendance and risk of childhood acute lymphoblastic leukaemia. *Br J Cancer*. 2002;86(9):1419-1424. doi:10.1038/sj.bjc.6600274
8. Hooper LV, Littman DR, Macpherson AJ. Interactions between the microbiota and the immune system. *Science*. 2012;336(6086):1268-1273. doi:10.1126/science.1223490
9. Tamburini S, Shen N, Wu HC, Clemente JC. The microbiome in early life: implications for health outcomes. *Nat Med*. 2016;22(7):713-722. doi:10.1038/nm.4142
10. Weingarden AR, Vaughn BP. Intestinal microbiota, fecal microbiota transplantation, and inflammatory bowel disease. *Gut Microbes*. 2017;8(3):238-252. doi:10.1080/19490976.2017.1290757
11. Korpela K, Salonen A, Vepsäläinen O, et al. Probiotic supplementation restores normal microbiota composition and function in antibiotic-treated and in caesarean-born infants. *Microbiome*. 2018;6:182. doi:10.1186/s40168-018-0567-4
12. Juul FE, Garborg K, Bretthauer M, et al. Fecal Microbiota Transplantation for Primary Clostridium difficile Infection. *N Engl J Med*. 2018;378(26):2535-2536. doi:10.1056/NEJMc1803103
13. DeFilipp Z, Peled JU, Li S, et al. Third-party fecal microbiota transplantation following allo-HCT reconstitutes microbiome diversity. *Blood Adv*. 2018;2(7):745-753. doi:10.1182/bloodadvances.2018017731
14. Routy B, Le Chatelier E, Derosa L, et al. Gut microbiome influences efficacy of PD-1-based immunotherapy against epithelial tumors. *Science*. 2018;359(6371):91-97. doi:10.1126/science.aan3706
15. Vatanen T, Franzosa EA, Schwager R, et al. The human gut microbiome in early-onset type 1 diabetes from the TEDDY study. *Nature*. 2018;562(7728):589-594. doi:10.1038/s41586-018-0620-2
16. Rook G a. W. 99th Dahlem conference on infection, inflammation and chronic inflammatory disorders: darwinian medicine and the "hygiene" or "old friends" hypothesis. *Clin Exp Immunol*. 2010;160(1):70-79. doi:10.1111/j.1365-2249.2010.04133.x
17. Bridgman SL, Kozyrskyj AL, Scott JA, Becker AB, Azad MB. Gut microbiota and allergic disease in children. *Ann Allergy Asthma Immunol Off Publ Am Coll Allergy Asthma Immunol*. 2016;116(2):99-105. doi:10.1016/j.anai.2015.10.001
18. Greaves M. A causal mechanism for childhood acute lymphoblastic leukaemia. *Nat Rev Cancer*. 2018;18(8):471-484. doi:10.1038/s41568-018-0015-6
19. Bach J-F. The hygiene hypothesis in autoimmunity: the role of pathogens and commensals. *Nat Rev Immunol*. 2018;18(2):105-120. doi:10.1038/nri.2017.111
20. The EndNote Team. *EndNote*. Clarivate; 2013.
21. Bramer WM, Giustini D, de Jonge GB, Holland L, Bekhuis T. De-duplication of database search results for systematic reviews in EndNote. *J Med Libr Assoc JMLA*. 2016;104(3):240-243. doi:10.3163/1536-5050.104.3.014
22. *Covidence Systematic Review Software*. Veritas Health Innolvation www.covidence.org
